# Supplementary material for: Characterization of Philadelphia-like Pre-B Acute Lymphoblastic Leukemia: Experiences in Mexican Pediatric Patients
Source: Int J Mol Sci. 2022 Aug 24;23(17):9587. doi: 10.3390/ijms23179587 (PMC9455471; doi:10.3390/ijms23179587)
Supplement: Supplementary file 1 [file ijms-23-09587-s001.zip › ijms-1818043-supplementary.pdf]

Table S1. Clinical and laboratory characteristics of 104 pre-B ALL children

ID	Gender	Age (years)	Risk Stratification	CNS involvement	Testicular involvement	WBC count x10^9/L	*CRLF2* Overexpression	*CRLF2* Rearrangement	*IGH* Breakage	*CRLF2* Extra copies	Mutations	*IKZF1* Lesion	iAMP21	GEP (%)	Positivity to surrogate markers
ALL001	MALE	15	HIGH	YES	NO	700	POS	-	-	-	*CRLF2, NRASx2*	Ik6	-	57**	-
ALL002	FEMALE	13	HIGH	NO	NO	428000	POS	*IGH::CRLF2*	-	-	*JAK2, KRAS, PAX5x2*	-	-	83	-
ALL003	FEMALE	10	HIGH	NO	NO	62500	POS	*P2RY8::CRLF2*	-	-	-	-	POS	100**	-
ALL004	MALE	NA	NA	NA	NA	NA	POS	*P2RY8::CRLF2*	-	-	-	-	-	67	-
ALL005	MALE	8	HIGH	YES	NO	6640	POS	*P2RY8::CRLF2*	-	-	-	-	-	71**	-
ALL006	FEMALE	15	HIGH	NO	NO	160600	POS	-	-	-	-	deletion	-	83	-
ALL007	MALE	16	HIGH	NO	NO	155900	POS	-	-	*CRLF2*x3	-	deletion	-	67	-
ALL008	MALE	12	HIGH	YES	NO	161100	POS	-	POS	-	-	Ik6	-	100**	-
ALL009	FEMALE	8	STANDARD	NO	NO	3200	POS	*IGH::CRLF2*	-	-	*JAK1*	-	-	83	-
ALL010	MALE	14	HIGH	YES	YES	108000	POS	*IGH::CRLF2*	-	-	*JAK2, KRAS*	-	-	83	-
ALL011	FEMALE	5	HIGH	NO	NO	16200	POS	*P2RY8::CRLF2*	-	-	*JAK2*	Ik6	-	83	-
ALL012	FEMALE	6	STANDARD	NO	NO	3400	POS	*P2RY8::CRLF2*	-	-	-	-	-	83	-
ALL013	FEMALE	3	HIGH	NO	NO	10800	POS	-	-	-	*FLT3*	-	-	33	pCrkl
ALL014	MALE	9	VERY HIGH	NO	NO	80020	POS	-	-	Hyperdiploidy	-	-	-	NA	-
ALL015	FEMALE	7	STANDARD	NO	NO	5300	POS	-	-	Hyperdiploidy	-	Ik6	-	67	-
ALL016	FEMALE	7	HIGH	YES	NO	1700	POS	*P2RY8::CRLF2*	-	-	-	-	-	NA	-
ALL017	FEMALE	10	HIGH	NO	NO	3500	POS	-	-	-	*SH2B3x2*	Ik6	POS	83	-
ALL018	MALE	11	HIGH	NO	NO	16800	POS	*P2RY8::CRLF2*	-	-	-	Ik6	-	71	-
ALL019	FEMALE	3	STANDARD	NO	NO	5980	POS	*IGH::CRLF2*	-	-	-	-	-	83	-
ALL020	MALE	1	HIGH	NO	NO	67800	POS	-	-	Hyperdiploidy	*NRAS*	Ik6	-	14	-

ALL021	MALE	4	HIGH	YES	NO	7390	POS	*P2RY8::CRLF2*	-	-	-	Ik6	-	NA	-
ALL022	FEMALE	5.5	STANDARD	NO	NO	1300	POS	*IGH::CRLF2*	-	-	*NRAS*	Ik6	-	50	-
ALL023	FEMALE	9	HIGH	YES	NO	4000	POS	*P2RY8::CRLF2*	-	-	-	-	-	67	-
ALL024	MALE	16	HIGH	NO	NO	1200	POS	*IGH::CRLF2*	-	-	-	Ik6	-	50	-
ALL025	MALE	10	HIGH	YES	NO	2000	POS	*P2RY8::CRLF2*	-	-	-	-	-	71	-
ALL026	FEMALE	4	HIGH	NO	NO	2100	POS	*P2RY8::CRLF2*	-	-	-	Ik6	-	50	pStat5/pCrkl
ALL027	FEMALE	16	HIGH	YES	NO	34700	POS	-	-	-	*KRAS*	Ik6	-	16	-
ALL028	MALE	4	HIGH	NO	NO	157500	POS	-	-	-	-	-	-	16	-
ALL029	MALE	6	STANDARD	NO	NO	3400	POS	*P2RY8::CRLF2*	-	-	*NF1*	-	-	33	-
ALL030	FEMALE	NA	NA	NA	NA	NA	POS	-	-	Hyperdiploidy	-	Ik6	-	16	-
ALL031	MALE	3	STANDARD	NO	NO	1800	POS	-	-	-	-	-	-	50	-
ALL032	MALE	12	HIGH	YES	NO	14200	POS	*P2RY8::CRLF2*	-	-	-	-	-	16	pStat5/pCrkl
ALL033	MALE	11	HIGH	NO	NO	200900	POS	-	POS	-	*NF1*	Ik6	-	100	-
ALL034	MALE	4	HIGH	YES	NO	420	POS	-	-	Hyperdiploidy	-	-	-	57	-
ALL035	FEMALE	NA	NA	NA	NA	NA	POS	-	-	-	-	-	-	50	-
ALL036	FEMALE	NA	NA	NA	NA	NA	POS	*IGH::CRLF2*	-	-	*NRAS, USP9X*	Ik6	-	67	pStat5/pCrkl/pErk
ALL037	FEMALE	4	VERY HIGH	NO	NO	90800	POS	*IGH::CRLF2*	-	-	-	Ik6	-	67	-
ALL038	MALE	5	HIGH	YES	NO	11630	POS	*P2RY8::CRLF2*	-	-	-	-	-	33	-
ALL039	MALE	9.8	NA	NA	NA	84 200	POS	*P2RY8::CRLF2*	-	-	*PTPN11, PAX5*	-	-	50	pCrkl
ALL040	FEMALE	14	HIGH	NO	NO	86500	POS	-	-	-	*NRAS*	deletion	-	100	pCrkl/pErk
ALL041	FEMALE	3	HIGH	NO	NO	33000	POS	*P2RY8::CRLF2*	-	-	-	Ik6	POS	33	-
ALL042	MALE	NA	NA	NA	NA	NA	POS	-	-	-	*BRAF*	-	-	50	-
ALL043	FEMALE	4	VERY HIGH	NO	NO	5930	POS	-	-	Hyperdiploidy	-	Ik6	-	50	-
ALL044	FEMALE	16	HIGH	NO	NO	12790	POS	-	-	-	-	-	-	16	-

ALL045	MALE	15	HIGH	NO	NO	3200	POS	*IGH::CRLF2*	-	-	*NRAS, IKZF1x2, PAX5*	mutations	-	33	pStat5/pCrkl
ALL046	FEMALE	4	HIGH	NO	NO	10600	POS	*IGH::CRLF2*	-	-	*KRASx2, CSF1R*	Ik6	-	50	pCrkl
ALL047	MALE	1	HIGH	NO	NO	20600	POS	-	-	-	-	-		67	-
ALL048	MALE	6	HIGH	NO	NO	17400	POS	*P2RY8::CRLF2*	-	-	-	Ik6	POS	67	-
ALL049	FEMALE	13	HIGH	YES	NO	4600	POS	-	-	-	-	-	-	33	-
ALL050	MALE	2	HIGH	NO	NO	1800	POS	*P2RY8::CRLF2*	-	-	*KRAS*	Ik6	POS	83	-
ALL051	MALE	2	HIGH	YES	NO	97000	POS	*P2RY8::CRLF2*	-	-	-	Ik6	-	16	-
ALL052	FEMALE	10	HIGH	NO	NO	3700	POS	-	-	-	-	Ik6	-	100	-
ALL053	FEMALE	9	HIGH	NO	NO	9400	POS	*P2RY8::CRLF2*	-	-	-	Ik6	-	71	-
ALL054	MALE	12	HIGH	NO	NO	45000	POS	*P2RY8::CRLF2*	-	-	-	Ik6	POS	67	-
ALL055	FEMALE	4	HIGH	NO	NO	26100	POS	-	POS	-	-	Ik6	-	83	-
ALL056	FEMALE	13	VERY HIGH	NO	NO	16800	POS	*P2RY8::CRLF2*	-	-	-	Ik6	-	33	-
ALL057	MALE	6	HIGH	NO	NO	2100	POS	-	-	-	-	Ik6	-	67	pCrkl
ALL058	FEMALE	2	STANDARD	NO	NO	11000	NEG	-	-	-	-	-	-	57	-
ALL059	MALE	2	HIGH	YES	NO	48300	NEG	-	-	-	-	-	-	0	-
ALL060	MALE	4	STANDARD	NO	NO	6100	NEG	-	-	-	-	Ik6	-	16	pStat5
ALL061	MALE	4	STANDARD	NO	NO	3100	NEG	-	-	-	-	Ik6	-	33	pCrkl
ALL062	MALE	6	STANDARD	NO	NO	2800	NEG	-	-	-	-	-	-	67	-
ALL063	FEMALE	4	STANDARD	NO	NO	3000	NEG	-	-	-	-	-	-	0	-
ALL064	FEMALE	4	STANDARD	NO	NO	600	NEG	*P2RY8::CRLF2*	-	-	-	Ik6	-	67	-
ALL065	MALE	2	HIGH	NO	NO	60000	NEG	*P2RY8::CRLF2*	-	-	-	Ik6	POS	57	-
ALL066	FEMALE	2	HIGH	NO	NO	358900	NEG	-	-	-	*KRAS*	Ik6	-	0	-
ALL067	MALE	14	HIGH	NO	NO	400	NEG	-	-	-	*PTPN11*	-	-	16	-

ALL068	MALE	14	HIGH	NO	NO	51400	NEG	-	-	-	*NRAS*	-	-	0	-
ALL069	MALE	5	HIGH	NO	NO	5200	NEG	-	-	-	-	Ik6	-	50	-
ALL070	MALE	3	HIGH	NO	NO	83600	NEG	-	-	-	*NF1*	Ik6	-	0	-
ALL071	MALE	7	HIGH	NO	NO	6000	NEG	*P2RY8::CRLF2*	-	-	*NRAS*	Ik6	POS	16	-
ALL072	FEMALE	2	HIGH	NO	NO	56000	NEG	-	-	-	*PAX5*	-	-	50	-
ALL073	FEMALE	8	HIGH	NO	NO	174600	NEG	-	-	Hyperdiploidy	-	Ik6	-	67	-
ALL074	FEMALE	9	HIGH	NO	NO	1100	NEG	*P2RY8::CRLF2*	-	-	-	-	-	50	-
ALL075	FEMALE	13	HIGH	NO	NO	3000	NEG	-	-	-	-	Ik6	-	33	-
ALL076	FEMALE	4	STANDARD	NO	NO	2600	NEG	-	-	Hyperdiploidy	-	-	-	33	-
ALL077	MALE	16	HIGH	NO	NO	184000	NEG	-	-	-	-	Ik6	-	67	-
ALL078	FEMALE	8	HIGH	NO	NO	73700	NEG	-	-	-	-	Ik6	-	67	-
ALL079	FEMALE	2	STANDARD	NO	NO	5130	NEG	*P2RY8::CRLF2*	-	-	-	Ik6	-	50	-
ALL080	MALE	14	HIGH	NO	NO	3100	NEG	*P2RY8::CRLF2*	-	-	-	-	-	50	-
ALL081	MALE	4	HIGH	NO	NO	29000	NEG	*P2RY8::CRLF2*	-	-	-	-	-	67	-
ALL082	FEMALE	14	HIGH	NO	NO	40400	NEG	*P2RY8::CRLF2*	-	-	-	Ik6	-	16	-
ALL083	FEMALE	9	STANDARD	NO	NO	17500	NEG	-	-	-	-	-	-	28	-
ALL084	MALE	13	HIGH	NO	NO	6700	NEG	*P2RY8::CRLF2*	-	-	-	Ik6	-	33	-
ALL085	FEMALE	5	HIGH	NO	NO	7700	NEG	*P2RY8::CRLF2*	-	-	-	Ik6	POS	16	-
ALL086	FEMALE	2	HIGH	NO	NO	1200	NEG	-	-	-	-	Ik6	-	50	-
ALL087	FEMALE	12	HIGH	NO	NO	1600	NEG	-	-	-	-	Ik6	-	33	-
ALL088	MALE	NA	NA	NA	NA	NA	NEG	-	-	-	-	-	-	42	-
ALL089	MALE	2	STANDARD	NO	NO	4500	NEG	-	-	-	-	-	-	0	-
ALL090	MALE	8	HIGH	NO	NO	4300	NEG	-	-	-	-	Ik6	-	0	-
ALL091	FEMALE	5	STANDARD	NO	NO	20200	NEG	-	-	-	-	-	-	16	-
ALL092	MALE	2	STANDARD	NO	NO	2100	NEG	-	-	-	-	-	-	0	-

ALL093	MALE	2	HIGH	YES	NO	21000	NEG	-	-	-	-	Ik6	-	0	-
ALL094	FEMALE	13	HIGH	NO	NO	15800	NEG	-	-	-	-	-	-	0	-
ALL095	MALE	12.6	HIGH	NO	NO	53800	NEG	-	-	-	-	Ik6	-	0	-
ALL096	MALE	0.9	STANDARD	NO	NO	25980	NEG	-	-	-	-	Ik6	-	16	-
ALL097	MALE	3	HIGH	YES	NO	2910	NEG	*P2RY8::CRLF2*	-	-	-	Ik6	-	33	-
ALL098	MALE	15	HIGH	NO	NO	16000	NEG	-	-	-	-	Ik6	-	42	-
ALL099	FEMALE	10	HIGH	YES	NO	9600	NEG	*P2RY8::CRLF2*	-	-	-	Ik6	POS	16	-
ALL100	MALE	14	HIGH	YES	NO	7000	NEG	*P2RY8::CRLF2*	-	-	-	-	POS	0	-
ALL101	MALE	5	HIGH	YES	NO	420	NEG	-	-	-	-	Ik6	-	0	-
ALL102	MALE	10	HIGH	YES	NO	17300	NEG	-	-	-	-	-	-	0	-
ALL103	MALE	9	VERY HIGH	NO	NO	2900	NEG	-	-	-	-	-	-	0	-
ALL104	FEMALE	3	STANDARD	NO	NO	3610	NEG	-	-	-	-	-	-	16	-

NA= no available

GEP= gene expression profile

** Positive to Reshmi's Ph-like profile

Table S2. Classification of the variants detected by NGS and Sanger sequencing

Gene	Position (hg19)	Mutation type	Nucleotide change	Protein change	Patient	VAF	ACMG Classification
CRLF2	chrX:1314966	missense variant	c.695T>G ^	p.Phe232Cys	ALL 001	0.38	Uncertain Significance.
JAK2	chr9:5078360	missense variant	c.2047A>G *	p.Arg683Gly	ALL 002	0.48	Likely Pathogenic
JAK1	chr1:65304236	missense variant	c.2879C>T	p.Pro960Leu	ALL 009	0.24	Likely Pathogenic
JAK3	chr19:17943694	missense variant	c.2395C>T	p.Arg799Cys	ALL 040	0.48	Uncertain Significance
SH2B3	chr12:111884622 -111884623	frameshift variant	c.799dup	p.Glu267GlyfsTer4	ALL 017	0.87	Pathogenic
	chr12:111884624	missense variant	c.800A>G	p.Glu267Gly	ALL 017	0.91	Likely Pathogenic
KRAS	chr12:25398284	missense variant	c.35G>T *	p.Gly12Val	ALL 046	0.21	Pathogenic
	chr12:25398285	missense variant	c.34G>A *	p.Gly12Ser	ALL 010	0.38	Pathogenic
	chr12:25398284	missense variant	c.35G>A *	p.Gly12Asp	ALL 046	0.20	Pathogenic
					ALL 066	0.42	
	chr12:25398281	missense variant	c.38G>A*	p.Gly13Asp	ALL 050	0.38	Pathogenic

	chr12:25378562	missense variant	c.436G>A	p.Ala146Thr	ALL 002	0.18	Pathogenic
	chr12:25380285	missense variant	c.173C>T	p.Thr58Ile	ALL 027	0.47	Pathogenic
NRAS	chr1:115256530	missense variant	c.181C>A	p.Gln61Lys	ALL 020	0.33	Pathogenic
	chr1:115258745	missense variant	c.37G>C *	p.Gly13Arg	ALL 036	0.33	Pathogenic
	chr1:115256521	missense variant	c.190T>A	p.Tyr64Asn	ALL 040	0.20	Pathogenic
	chr1:115258747	missense variant	c.34G>A *	p.Gly12Ser	ALL 068	0.38	Pathogenic
					ALL 071	0.31	
	chr1:115258744	missense variant	c.38G>A *	p.Gly13Asp	ALL 045	0.37	Pathogenic
					ALL 022	0.45	
	chr1:115258747	missense variant	c.35G>T *	p.Gly12Val	ALL 001	0.54	Pathogenic

	chr1:115258747	missense variant	c.35G>A *	p.Gly12Asp	ALL 001	0.10	Pathogenic
NF1	chr17:29556187	missense variant	c.2554C>T	p.Leu852Phe	ALL 033	0.45	Likely Pathogenic
	chr17:29664504	stop gained	c.6546C>A	p.Tyr2182Ter	ALL 070	0.31	Likely Pathogenic
	chr17:29670036	stop gained	c.7072G>T	p.Glu2358Ter	ALL 029	0.97	Likely Pathogenic
	chr17:29509608	missense variant	c.813C>G	p.Ile271Met	ALL 027	0.25	Uncertain Significance
PTPN11	chr12:112888156	missense variant	c.172A>T	p.Asn58Tyr	ALL 067	0.50	Pathogenic
	chr12:112888165	missense variant	c.181G>T	p.Asp61Tyr	ALL 039	0.35	Pathogenic
BRAF	chr7:140453132	missense variant	c.1803A>C	p.Lys601Asn	ALL 042	0.47	Pathogenic
FLT3	chr13:28602329	missense variant	c.2039C>T	p.Ala680Val	ALL 013	0.46	Likely Pathogenic
	chr13:28608309	missense variant	c.1747G>A	p.Gly583Ser	ALL 049	0.33	Uncertain Significance
	chr13:28609667	missense variant	c.1562G>C	p.Gly521Ala	ALL 067	0.49	Uncertain Significance
	chr13:28608338	missense variant	c.1718A>G	p.Glu573Gly	ALL 042	0.46	Uncertain Significance

	chr13:28623626	missense variant	c.931C>T	p.Arg311Trp	ALL 019	0.48	Uncertain Significance
CSF1R	chr5:149441113	missense variant	c.1799C>T	p.Thr600Met	ALL 046	0.50	Likely Pathogenic
	chr5:149460542	missense variant	c.95T>G	p.Val32Gly	ALL 050	0.49	Likely Benign
	chr5:149449827	missense variant	c.1237G>A	p.Gly413Ser	ALL 001	0.49	Benign
ABL2	chr1:179077709	missense variant	c.2693G>A	p.Arg898Gln	ALL 011	0.50	Likely Benign
PDGFRA	chr4:55127448	missense variant	c.236G>A	p.Gly79Asp	ALL 068	0.49	Benign
NTRK3	chr15:88799284	missense variant	c.101C>A	p.Ala34Glu	ALL 040	0.49	Uncertain Significance
SSBP2	chr5:80736449	missense variant	c.880A>G	p.Met294Val	ALL 068	0.52	Uncertain Significance
IKZF1	chr7:50367250-50367251	frameshift variant	c.50_51insGTAAGCG	(p.Ser17ArgfsTer2)	ALL 045	0.33	Pathogenic
	chr7:50467673-50467674	frameshift variant	c.907_908insGGCCTGGGGGCCT	(p.Asn303ArgfsTer190)	ALL 045	0.41	Pathogenic
ETV6	chr12:12022496	missense variant	c.602T>C	p.Leu201Pro	ALL 020	0.5	Benign

PAX5	chr9:36966684-36966687	frameshift variant	c.639_642del	p.Leu214ArgfsTer63	ALL 002	0.3	Pathogenic
	chr9:36966690-36966702	frameshift variant	c.624_636del	p.Pro209ArgfsTer65	ALL 002	0.30	Pathogenic
	chr9:37020768-37020769	frameshift variant	c.76dup	p.Val26GlyfsTer49	ALL 072	0.38	Pathogenic
	chr9:37015009	missense variant	c.395T>C	p.Val132Ala	ALL 077	0.90	Uncertain significance
	chr9:36882049	missense variant	c.964G>A	p.Ala322Thr	ALL 004	0.52	Benign
					ALL 001	0.47	
	chr9:37015160	missense variant	c.244G>A	p.Val82Ile	ALL 045	0.45	Benign
					ALL 057	0.97	
	chr9:37015165	missense variant	c.239C>G	p.Pro80Arg	ALL 045	0.38	Pathogenic
					ALL 039	0.46	
USP9X	chrX:41091705	missense variant	c.7641A>C	p.Arg2547Ser	ALL 036	0.95	Uncertain Significance
	chrX:41091709	missense variant	c.7645G>A	p.Gly2549Ser	ALL 036	0.95	Uncertain Significance

	chrX:41091710	missense variant	c.7646G>A	p.Gly2549Asp	ALL 036	0.94	Uncertain Significance
	chrX:41091707-41091708	frameshift variant	c.7644_7645insTTACT	p.Gly2549LeufsTer92	ALL 036	0.90	Pathogenic

^ Detected by Sanger sequencing. * Detected by NGS and confirmed by Sanger sequencing. VAF: Variant allele fraction.

Table S3. Primers and probe sets for Gene expression profile (GEP) and Sanger sequencing

GEP			
Gene	Forward 5´- 3´	Reverse 5´- 3´	Probe*
CRLF2 NM_022148	AGCGACTGGTCAGAGGTGA	AATTTGGACAGCTTTGGTTTG	#53
TSPN7 NM_004615	CATCTACTCCTTCGTCTTCTGGA	AGTAAGTTTGCCCCAGACTCC	#18
IGJ NM_144646	TGTGGAGAGAAACATCCGAATTA	ATGGTGAGGTGGGATCAGAG	#6
PON2 NM_000305	TCCACACTGCCACCTGATTA	AGCCAGACCATTGGGAAGTAT	#21
SEMA6A NM_001300780	TGGACACCAGTTCCTGATGA	TCATTGGAGGTTGCATATCTTT C	#88

BMPR1 NM_001203	TTTCATGCCTTGTTGATAAAGGT	GCTTGTTTAACTTTTTGTTTCCT CTC	#21
MUC4 NM_018406	CTCCTTCCGCATGTGGTC	TGTTGAGCCTGTTGAGGTGA	#74
GUSβ NM_000181	CGCCCTGCCTATCTGTATTC	TCCCCACAGGGAGTGTGTGTA G	#57
	Sanger sequencing		
*CRLF2***	CCGCCTTTTCATTTTGTTTC	CACAGACATTGTGCAAGCAG	
*JAK2***	ATGCCTCCAAATTATTATACTATC A	ATCACCTCACAGTCCATGGTTAT	
KRAS	TTTGTGAGGGTGTGCTACAGG	TGATGTCACAATACCAAGAAACCC	
NRAS	GCTTTAAAGTACTGTAGATGTGG C	AAAGATGATCCGACAACTGAGAG	

*Universal Probe Library System Roche®, ** Yoda et al., 2010 [43].
